# Reframing Benign Prostatic Hyperplasia as a Cardiometabolic Condition: Should BPH Be Considered a Metabolic Disease?

**DOI:** 10.31083/RCM52887

**Published:** 2026-07-17

**Authors:** Ioanna Dimitriadou, Michael Samarinas

**Affiliations:** ^1^Department of Nursing, University of Thessaly, 41500 Larisa, Greece; ^2^Urology Department, Aristotle University of Thessaloniki, Papageorgiou General Hospital, 56429 Thessaloniki, Greece

We read with great interest the growing body of literature exploring the intersection between the metabolic syndrome, cardiovascular disease (CVD), and benign prostatic hyperplasia (BPH), and we wish to highlight the increasing evidence that BPH should no longer be regarded solely as an age-related urological disorder, but rather as a manifestation of systemic cardiometabolic dysfunction.

We propose that benign prostatic hyperplasia (BPH) should be reconsidered as a manifestation of systemic cardiometabolic dysfunction, and potentially redefined as a metabolic disease. Increasing evidence demonstrates a strong association between BPH/lower urinary tract symptoms (LUTS) and cardiovascular risk factors, including metabolic syndrome, insulin resistance, and endothelial dysfunction. While causality remains to be established, these findings suggest that BPH may represent an early clinical marker of systemic vascular disease rather than an isolated urological condition. Recent large-scale population studies further support a link between BPH and increased cardiovascular risk, reinforcing the concept that BPH may reflect systemic vascular pathology rather than an isolated urological condition [1].

BPH remains one of the most prevalent conditions affecting aging males. Histological evidence of BPH is present in nearly 50% of males by the sixth decade of life and up to 80% by the ninth decade. Traditionally, BPH has been viewed primarily through the lens of androgenic stimulation and aging. However, emerging data suggest that this paradigm is incomplete [2,3]. Mounting evidence indicates that the pathogenesis and progression of BPH are strongly associated with cardiovascular and metabolic risk factors, including obesity, hypertension, insulin resistance, dyslipidemia, and chronic systemic inflammation [4].

The metabolic syndrome has consistently been associated with both increased prevalence and severity of BPH/LUTS, as confirmed by recent large cohort studies and meta-analyses [5]. Several epidemiological studies have demonstrated that men with metabolic syndrome exhibit significantly larger prostate volumes, worse symptom scores, and more rapid disease progression compared with metabolically healthy individuals [6,7]. In a large historical cohort of over 130,000 males, metabolic syndrome and unhealthy lifestyle factors were independently associated with BPH requiring treatment [6]. Similarly, multicenter prospective data have shown that the metabolic profile directly influences benign prostatic enlargement [7]. Higher overall cardiovascular health scores have been associated with a significantly lower risk of BPH, supporting the role of systemic metabolic health in disease prevention [8].

The mechanistic relationship between BPH and cardiovascular disease appears multifactorial (Fig. 1). Chronic low-grade inflammation, a hallmark of both atherosclerosis and metabolic syndrome, likely serves as a common pathogenic substrate, with recent studies suggesting that inflammatory and metabolic biomarkers partially mediate the association between metabolic syndrome and BPH [2,9]. Prostatic inflammatory infiltrates have been identified in the majority of BPH specimens and correlate with larger gland volume and worse LUTS. Inflammatory cytokines such as interleukin-6, tumor necrosis factor-α, and C-reactive protein may promote stromal proliferation, tissue remodeling, and fibromuscular hyperplasia within the prostate, mirroring the inflammatory vascular remodeling observed in cardiovascular disease [9].

**Fig. 1.**
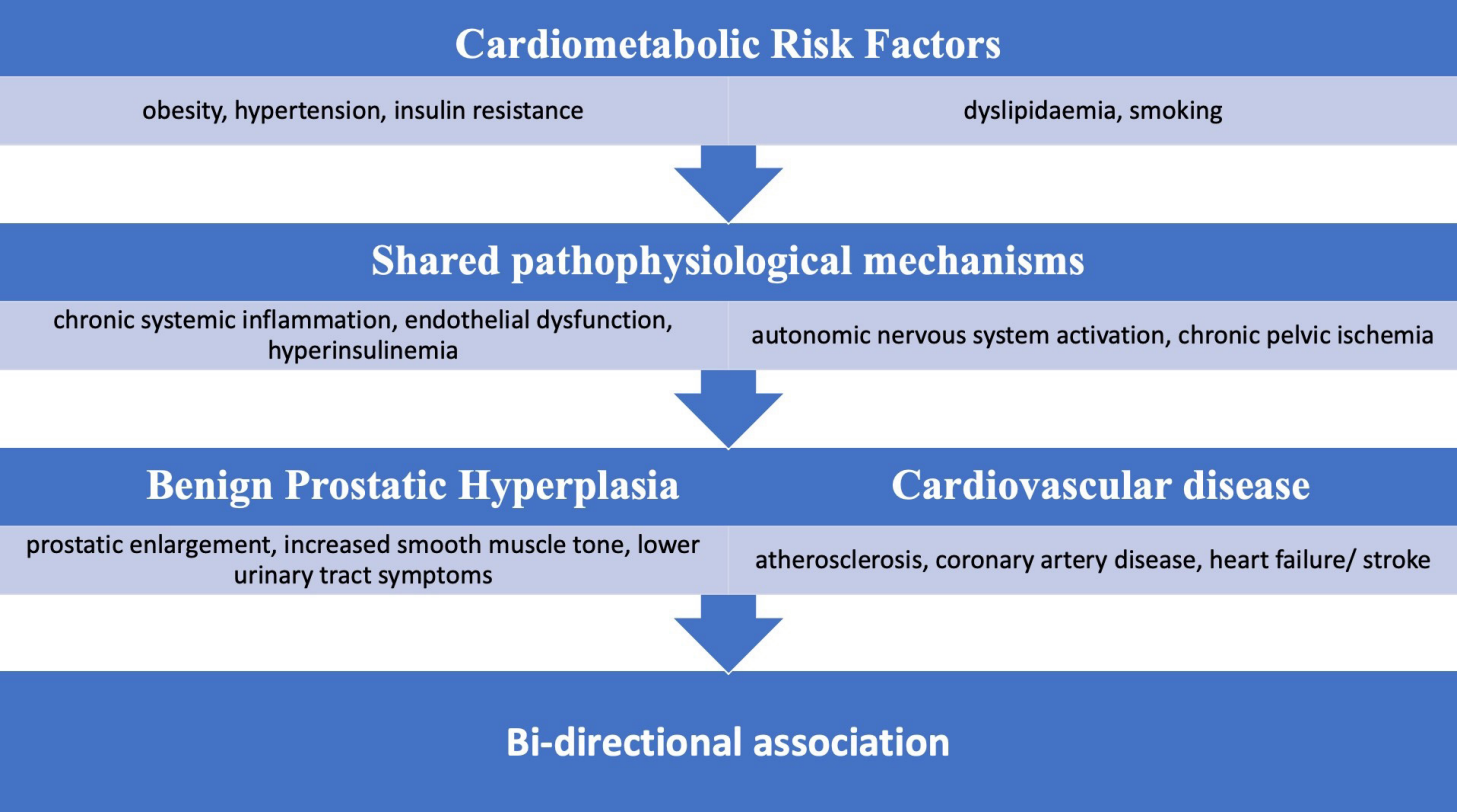
**Shared pathophysiological links between BPH and cardiovascular disease**. Proposed shared pathophysiological pathways linking benign prostatic hyperplasia (BPH) and cardiovascular disease (CVD). Common cardiometabolic risk factors contribute to overlapping biological mechanisms, including inflammation, endothelial dysfunction, and insulin resistance. Chronic pelvic ischemia is presented as a hypothetical pathway. BPH/lower urinary tract symptoms (LUTS) may represent an early clinical marker of systemic vascular disease.

Insulin resistance represents another plausible mechanistic link. Hyperinsulinemia may stimulate insulin-like growth factor pathways and sympathetic nervous system activity, thereby promoting prostatic smooth muscle tone and cellular proliferation [10]. In addition, endothelial dysfunction, central to cardiovascular disease, may contribute to chronic pelvic ischemia, which has been hypothesized to induce prostatic hypoxia, oxidative stress, and subsequent hyperplastic remodeling. This vascular hypothesis is supported by observational data and emerging genetic analyses suggesting a potential causal relationship between BPH and cardiovascular disease, although the evidence remains limited [11,12]. Males with hypertension are significantly more likely to present with severe LUTS and larger prostate volume than normotensive controls [12]. Endothelial dysfunction, central to cardiovascular disease, may contribute to chronic pelvic ischemia. This vascular hypothesis proposes that reduced pelvic blood flow induces prostatic hypoxia, oxidative stress, and subsequent hyperplastic remodeling [12]. While supported by observational associations between hypertension, atherosclerosis, and prostate enlargement, direct causal evidence remains limited, and this mechanism should be considered hypothesis-generating rather than definitive.

The association between BPH and cardiovascular disease may extend beyond shared risk factors. BPH/LUTS may itself represent an early clinical marker of systemic vascular disease. Several studies have demonstrated that males with moderate-to-severe LUTS have a higher incidence of major adverse cardiovascular events, independent of traditional cardiovascular risk factors [1]. This raises the possibility that LUTS/BPH may serve as a surrogate marker of endothelial dysfunction and generalized atherosclerotic burden. Recognition of this relationship has important clinical implications: the urologic consultation for LUTS may represent an underutilized opportunity for cardiovascular risk stratification and preventive intervention.

Therapeutically, this cardiometabolic perspective may reshape the management of BPH. Lifestyle modification and optimization of metabolic health may provide dual benefits for cardiovascular prevention and BPH progression. Weight reduction, exercise, improved glycemic control, and dietary interventions have all been associated with lower BPH prevalence and reduced severity of symptoms [6]. Furthermore, medications traditionally used for cardiovascular and metabolic disorders may possess beneficial effects on prostatic disease. The potential overlap between commonly used cardiometabolic therapies and their effects on BPH and cardiovascular outcomes is summarized in Table 1.

**Table 1.** **Potential overlap between cardiovascular/metabolic therapies and BPH outcomes**.

Drug class	Potential effects on BPH/LUTS	Cardiovascular effects	Key limitations/risks
Statins	Anti-inflammatory; may reduce prostate growth and LUTS progression	Lipid lowering; plaque stabilization; reduced CV events	Myopathy, liver enzyme elevation
ACE inhibitors/ARBs	Anti-fibrotic; possible improvement in LUTS	Blood pressure reduction; improved endothelial function	Hypotension, renal effects, hyperkalemia
PDE5 inhibitors	Improve LUTS; enhance pelvic perfusion	Improve endothelial function; exercise tolerance	Hypotension; contraindicated with nitrates
5α-reductase inhibitors	Reduce prostate volume; prevent progression	Uncertain; possible metabolic effects (exploratory)	Sexual dysfunction; unclear CV/metabolic impact

ACE, angiotensin converting enzyme; ARBs, angiotensin II receptor blockers; PDE5, phosphodiesterase 5 inhibitors; CV, cardiovascular; BPH, benign prostatic hyperplasia; LUTS, lower urinary tract symptoms.

For example, statins have demonstrated anti-inflammatory and antiproliferative effects on prostatic tissue, with emerging experimental and clinical evidence suggesting potential roles in BPH prevention, although findings remain inconclusive [13,14]. Angiotensin-converting enzyme inhibitors and angiotensin receptor blockers have also been implicated in ameliorating LUTS, possibly through anti-inflammatory and anti-fibrotic mechanisms. Experimental evidence further suggests that renin–angiotensin system modulation may attenuate prostatic hyperplasia [15].

Phosphodiesterase-5 inhibitors (PDE5i), already established in cardiovascular pharmacotherapy, occupy a unique position at the crossroads of urology and cardiology. Beyond improving LUTS in males with BPH, PDE5 inhibition may enhance endothelial function, improve pelvic perfusion, and provide cardioprotective metabolic effects. Their pleiotropic benefits reinforce the concept of shared pathophysiology between BPH and cardiovascular disease [3]. Concerns have been raised regarding potential metabolic consequences of 5α-reductase inhibitors, including insulin resistance and increased cardiovascular risk. However, current evidence remains inconsistent and largely exploratory. These findings should be interpreted with caution and should not currently alter standard clinical practice.

Nevertheless, several therapeutic considerations warrant caution. While 5α-reductase inhibitors remain a cornerstone in BPH management, concerns have been raised regarding their potential metabolic and cardiovascular effects, including possible associations with type 2 diabetes and vascular outcomes, although current evidence remains inconsistent and context-dependent [16]. Further prospective studies are needed to clarify whether BPH pharmacotherapy influences long-term cardiovascular outcomes.

However, the use of these agents in individuals without clear cardiovascular indications warrants caution. Potential risks, including hypotension, renal effects, and drug-to-drug interactions, should be carefully considered. Clinical decision-making should remain individualized, and these therapies should not be prescribed solely for presumed benefits on BPH without appropriate indications.

The implications of this evolving evidence are substantial. We propose that BPH/LUTS should be integrated into the broader framework of male cardiometabolic health rather than treated as an isolated urological entity. Cardiologists and primary care physicians should recognize LUTS as a potential marker of underlying metabolic and vascular disease, while urologists should consider cardiovascular screening in males presenting with progressive BPH, particularly those with early-onset or severe symptoms. From a practical standpoint, incorporation of LUTS assessment into cardiovascular risk evaluation may be feasible using validated tools such as the International Prostate Symptom Score (IPSS). Routine inquiry of LUTS using IPSS in males with hypertension, diabetes, or metabolic syndrome could serve as a simple and scalable screening strategy to identify individuals at higher cardiometabolic risk.

BPH represents a heterogeneous condition with variability in clinical presentation, histopathological features, and therapeutic response. Emerging evidence suggests that specific subgroups, particularly males with early-onset LUTS, obesity, insulin resistance, or overt metabolic syndrome, may exhibit a stronger link between prostatic disease and cardiometabolic dysfunction. Identification of such phenotypes may enable more precise risk stratification and targeted interventions.

It should be emphasized that the majority of existing evidence linking BPH with cardiometabolic disease arises from observational and epidemiological studies, which preclude definitive conclusions regarding causality [17]. While these associations are robust and consistent, further mechanistic investigations, including Mendelian randomization studies, experimental animal models, and interventional trials, are required to elucidate causal pathways and validate this proposed paradigm.

Future research should prioritize prospective longitudinal studies evaluating whether modification of cardiometabolic risk factors, particularly through lifestyle interventions such as weight loss, dietary changes, and physical activity, can alter the natural history of BPH. Randomized controlled trials assessing the bidirectional impact of BPH and cardiovascular therapies are also warranted.

In conclusion, contemporary evidence supports a paradigm shift in the understanding of BPH from a purely age-related androgen-dependent process to a complex cardiometabolic disorder intricately linked with cardiovascular disease. Recognizing BPH as part of systemic vascular and metabolic dysfunction may foster interdisciplinary collaboration, improve risk stratification, and ultimately enhance outcomes for aging males.

## Abbreviations

BPH, benign prostatic hyperplasia; CVD, cardiovascular disease; LUTS, lower urinary tract symptoms.
